# Senescence induces fundamental changes in the secretome of mesenchymal stromal cells (MSCs): implications for the therapeutic use of MSCs and their derivates

**DOI:** 10.3389/fbioe.2023.1148761

**Published:** 2023-05-09

**Authors:** Yesuf Siraj, Umberto Galderisi, Nicola Alessio

**Affiliations:** ^1^ Department of Experimental Medicine, University of Campania, Naples, Italy; ^2^ Department of Medical Laboratory Sciences, School of Health Sciences, College of Medicine and Health Sciences, Bahir Dar University, Bahir Dar, Ethiopia; ^3^ Department of Biology, Faculty of Science, Erciyes University, Kayseri, Türkiye; ^4^ Center for Biotechnology, Sbarro Institute for Cancer Research and Molecular Medicine, Temple University, Philadelphia, PA, United States

**Keywords:** mesenchymal stromal cells (MSC), aging, senescence, secretome, SASP

## Abstract

Mesenchymal stromal cells (MSCs) are a heterogeneous population containing multipotent adult stem cells with a multi-lineage differentiation capacity, which differentiated into mesodermal derivatives. MSCs are employed for therapeutic purposes and several investigations have demonstrated that the positive effects of MSC transplants are due to the capacity of MSCs to modulate tissue homeostasis and repair via the activity of their secretome. Indeed, the MSC-derived secretomes are now an alternative strategy to cell transplantation due to their anti-inflammatory, anti-apoptotic, and regenerative effects. The cellular senescence is a dynamic process that leads to permanent cell cycle arrest, loss of healthy cells’ physiological functions and acquiring new activities, which are mainly accrued through the release of many factors, indicated as senescence-associated secretory phenotype (SASP). The senescence occurring in stem cells, such as those present in MSCs, may have detrimental effects on health since it can undermine tissue homeostasis and repair. The analysis of MSC secretome is important either for the MSC transplants and for the therapeutic use of secretome. Indeed, the secretome of MSCs, which is the main mechanism of their therapeutic activity, loses its beneficial functions and acquire negative pro-inflammatory and pro-aging activities when MSCs become senescent. When MSCs or their derivatives are planned to be used for therapeutic purposes, great attention must be paid to these changes. In this review, we analyzed changes occurring in MSC secretome following the switch from healthy to senescence status.

## 1 Introduction

The process of senescence induces loss of cellular functions and is associated with permanent cell cycle exit. Senescence may be induced by the impaired activity of lysosomes and mitochondria, the accumulation of unrepaired or misrepaired DNA, presence of nonfunctional/proteins, the oxidation of cellular macromolecules (Campisi and D’adda Di Fagagna, 2007; Lopez-Otin et al., 2023). Senescence may have beneficial and negative consequences on health: it may promote organismal aging through impairment of tissue renewal and function; it may also have an anti-cancer effect since it can arrest cancer growth. Moreover, senescence may promote wound healing (Campisi and D’adda Di Fagagna, 2007; Kuilman and Peeper, 2009).

The senescence occurring in stem cells, such as those present in mesenchymal stromal cells, may have detrimental effects on health since it can undermine tissue homeostasis and repair. This issue must be carefully evaluated to better understand the physiological organismal aging and when stem cells are used for therapeutic purposes.

## 2 Mesenchymal stromal cells (MSCs)

### 2.1 What are MSCs?

MSCs are a heterogenous population containing multipotent adult stem cells with a multi-lineage differentiation capacity, which are commonly differentiated into mesodermal derivatives such as adipocytes, chondrocytes, and osteocytes (Muraglia et al., 2000). MSCs can be derived from various human or animal body tissues including bone marrow, adipose tissue, amniotic fluid from the mid-trimester of pregnancy, umbilical cord, human synovial fluid, and human fetal bone tissue of an early-termination pregnancy (Özcan et al., 2016; Alessio et al., 2018; Alessio et al., 2019a; Gnani et al., 2019; Kehl et al., 2019; Alessio et al., 2020; Ratushnyy et al., 2020; Vassilieva et al., 2020; Grigorieva et al., 2021; Kizilay Mancini et al., 2021; Kwon et al., 2021; Liao et al., 2021; Wang B. et al., 2022; Miura et al., 2022). Besides the multipotential differentiation capacity, MSCs play a key role in tissue homeostasis and regeneration through the secretion of hundreds of biologically active factors (cytokines, chemokines, and growth and survival factors), which have a paracrine and long-range action (Galderisi et al., 2022). MSCs are, nowadays, employed for therapeutic purposes (Galderisi and Giordano, 2014).

### 2.2 Isolation of MSCs and therapeutic potential

According to the minimal criteria for defining multipotent mesenchymal stromal cells by the International Society for Cell and Gene Therapy (ISCT), the isolation and characterization of MSCs for different clinical purposes must fulfill the following major benchmarks: they must be able to adhere to plastic well surfaces; express surface markers of CD90, CD73, and CD105; be unable to express CD45, CD34, CD14α or CD11b, CD79 or CD19, and HLA-DR surface molecules; and able to differentiate into adipocytes, chondroblasts, and osteoblasts (Dominici et al., 2006). Although these criteria are basically employed in several stem cell laboratories for the identification and purification of MSCs, non-clonal cultures of bone marrow stromal cells may contain variable percentages of multipotent stem cells where committed progenitor and differentiated cells can also be present (Muraglia et al., 2000; Galderisi U et al., 2014).

The self-renewal, *in vitro* expandability, multipotent differentiation capacity, being less prone to senescence, and non-immunogenic but still immunomodulatory properties of MSCs from various tissue sources have attracted researchers for their clinical applications in the field of regenerative diseases (Zhang et al., 2015; Alessio et al., 2018; Alessio et al., 2019b). MSCs are candidates for cell-based therapeutic strategies for various disease conditions including neurodegenerative disorders, where clinical stabilization of non-option Parkinsonism was observed for at least 6 months, improvements observed in cardiovascular diseases, perianal fistulas associated with Crohn’s disease, COVID-19, bone disorders, and cancers (Giordano et al., 2014; Canesi et al., 2016; Kastrup et al., 2017; Liu S. et al., 2020; Cheng et al., 2020; Hmadcha et al., 2020; Wang et al., 2021; Johnson et al., 2022; Shi et al., 2022; Sohrabi et al., 2022). A clinical trial on the safety and feasibility of adipose-derived stromal cells also demonstrated a safe and feasible treatment of ischemic heart diseases and heart failure (Kastrup et al., 2017).

However, several factors including the risk of tumorigenicity and immunosuppression makes the reputation of stem cell therapies come into question (Madrid et al., 2021). The other essential concerns are the biological, legal, and societal issues related to the use and application of human-derived stem cells (Moradi et al., 2019). Therapeutic use of MSCs also still needs inclusive characterization guidelines to accommodate various sources including bone marrow, adipose tissue, amniotic membrane, umbilical cord, and others to alleviate the regulatory gaps in stem cell-based therapies (Wright et al., 2021). Safety related issues related to cell-based therapies and their products shall be repeatedly tested before their actual therapeutic application for regenerative disorders (Lu et al., 2011). Hence, various legal and ethical barriers may hinder the full exploitation of stem cells in clinical medicine; the laws and standards may be applied to ensure ethical integrity in the clinical practices of stem cell therapy (Moradi et al., 2019).

### 2.3 The secretome of MSCs is the main player of their therapeutic activity

In recent years, several investigations have demonstrated that the positive effects of MSC transplants are due to the capacity of MSCs to modulate tissue homeostasis via the activity of their secretome (Galderisi et al., 2022). The administration of MSC-derived conditioned medium, either as direct application of MSC-derived whole lysates or extracellular vesicles or exosomes, are now alternative strategies to cell transplantation due to their anti-inflammatory, anti-apoptotic, and regenerative effects (Gudiño and Salas, 2021). Moreover, novel vehicles of MSC-derived products are also currently in consideration for the delivery of miRNAs in cancer therapy (Sohrabi et al., 2022). The changes occurring in the secretome composition of MSCs during senescence may have negative consequences on the body’s health due to the importance of healthy MSC secretome in tissue repair and homeostasis (Caplan and Dennis, 2006; Hsiao et al., 2012). When MSCs or their derivates have to be used for therapeutic purposes, great attention must be paid to these changes.

## 3 MSC cell-based or secretome/conditioned media-based therapeutics

Stem cell-based therapies can offer the optional potential to cure various kinds of disorders that are incurable or difficult to treat. However, the only stem cell-based products approved by the United States Food and Drug Administration (FDA) for clinical use consists of hematopoietic stem cells derived from cord blood (FDA, 2022). The hematopoietic stem cell-based therapy was approved for limited use in patients with disorders of the hematopoietic system.

In December 2014, the European Medicines Agency (EMA) recommended the first stem cell-based therapy trademarked as Holoclar, containing stem cells, for approval to treat moderate to severe limbal stem cell deficiency that can result in blindness (EMA, 2014). Later in June 2018, Holoclar, which contains corneal epithelial stem cells, sponsored by *Holostem Terapie Avanzate S. r.l. Modena, Italy*, was also granted an orphan drug designation by the United States Food and Drug Administration for the treatment of limb stem cell deficiency (FDA, 2018).

The United States FDA and EMA are still cautious of the applications of stem cell product therapy, and both warned against using unapproved and unproven cell-based therapies, which may not be safe and effective. The EMA raised a crucial concern in that patients using unproven or unregulated cell-based therapies have reportedly suffered serious, sometimes fatal, side effects including infections, unwanted immune reactions, tumor formation, loss of vision, and bleeding in the brain (EMA, 2014).

A total of 1,179 MSC cell-based clinical trials (CTs) at different phases (I–III) were registered in the www.ClinicalTrials.gov database as of 15 November 2022, using a search term of “mesenchymal stem cell therapy” for the treatment of various conditions such as diabetic nephropathy, hemorrhagic stroke, hemophilia A and B, rheumatoid arthritis, bronchopulmonary dysplasia, autoimmune disorders, ulcerative colitis, acute myocardial infarction, COVID-19, and many other conditions. Currently, an increased number of registered clinical trials and varieties of diseases/conditions were observed compared to the previous records, where 493 CTs and 767 CTs were registered as of 15 June 2015 (Squillaro et al., 2016) and January 2020 (Liu J. et al., 2020), respectively.

**FIGURE 1:**
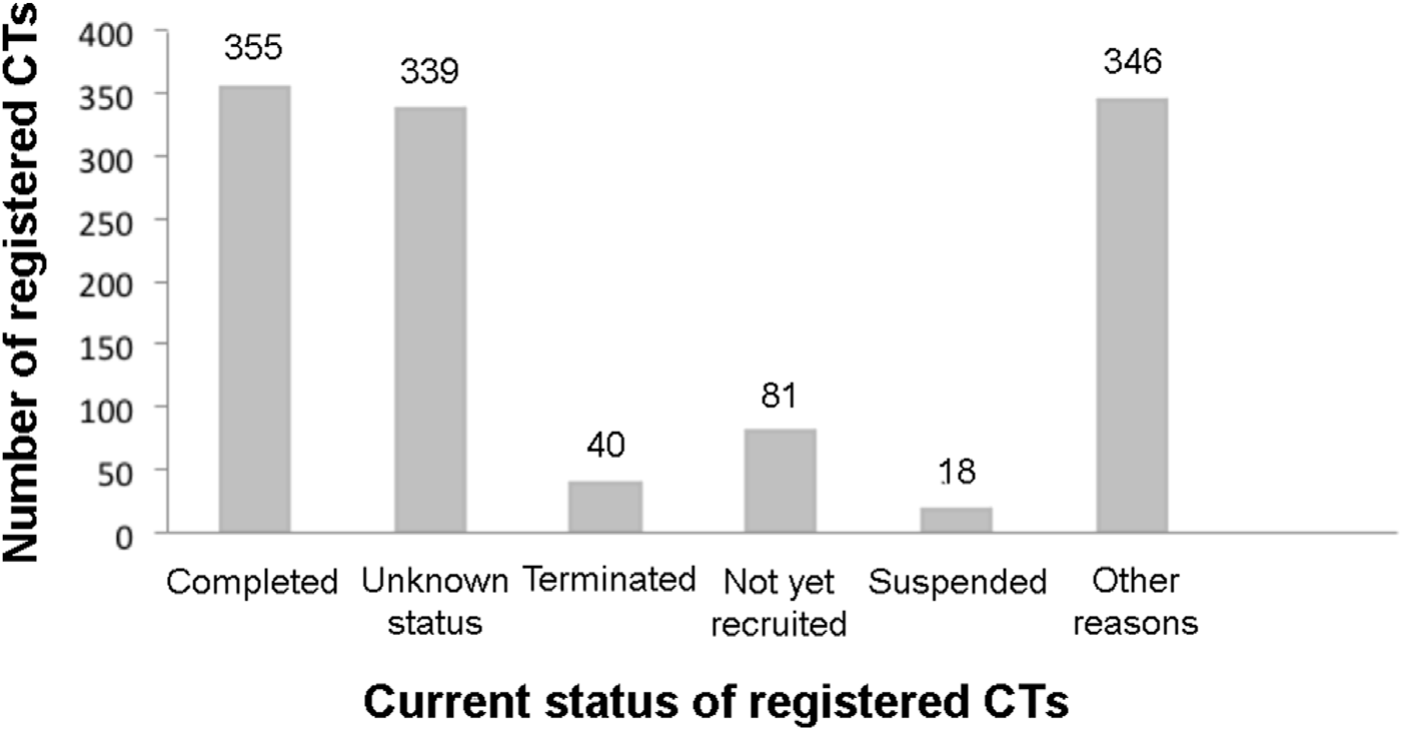
Current registered clinical trials of MSC based therapy. Data were retrieved from www.ClinicalTrial.org accessed on 15 November 2022.

About 30% of the total registered stem cell-based CTs were completed while 40 and 18 registered CTs were on terminated and suspended status, respectively (Figure 1), due to several reasons including the necessity of major revisions of the protocol, recruitment difficulties, inclusion defaults, availability of the vaccine, COVID-19 pandemic, political pressure, updates of NIH operational issues, low predictive probability of achieving postulated results, safety and precaution reasons and other unlisted explanations. The rest of the registered stem cell based CTs were under recruiting, active but not yet recruiting, unknown status, and some other status.

**FIGURE 2:**
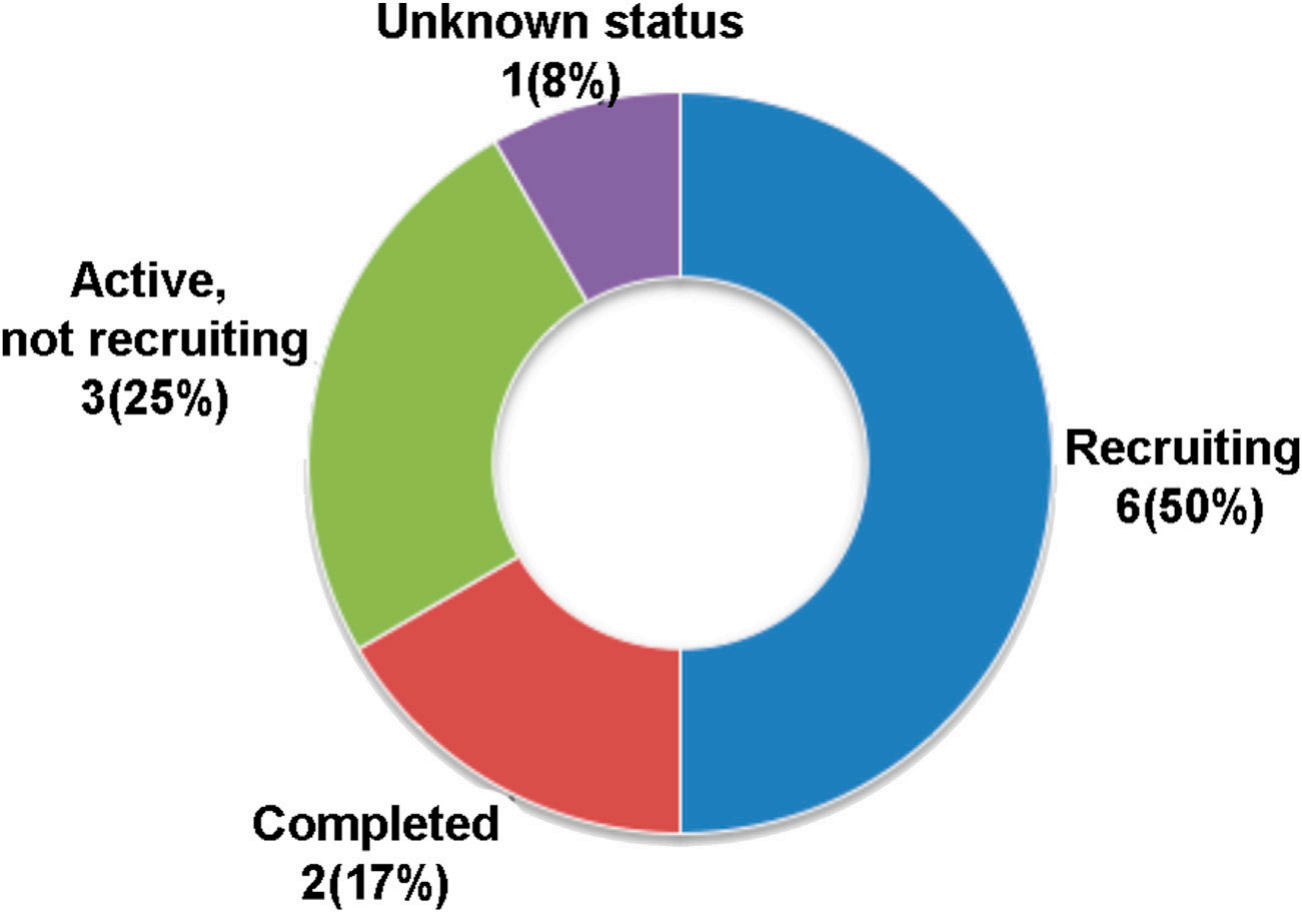
Current registered clinical trials of MSC secretome or conditioned media-based therapy. Data were retrieved from www.ClinicalTrial.org accessed on 15 November 2022.

MSC secretome or conditioned media-based therapy is also a recently introduced therapeutic strategy although the number of registered clinical trials in this category is much less than whole cell-based stem cell therapy. Only 12 registered MSC secretome/conditioned media therapy clinical trials were retrieved from the www.ClinicalTrials.org database, using a search term of “MSC secretome therapy” at the different statuses of complete 2 (17%), recruiting 6 (50%), active but not recruiting 3 (25%), and unknown status 1 (8%) (Figure 2).

The MSC secretome or conditioned medium-based therapy registered CTs were intended for the treatment of nasopharyngeal cancer, COVID-19, polycystic ovary syndrome or infertility, knee osteoarthritis, skin aging, chronic ulcers, ischemic stroke, corneal defect, and Keloid scars. The MSC secretome or conditioned medium-based therapy clinical trials are based on the fact that this alternative strategy may allow the direct application, easy distribution, and access to tissues of soluble secreted active molecules of MSCs, though getting a sufficient volume of secretome/conditioned media *in vitro* has not yet been resolved (Gudiño and Salas, 2021; Wang B. et al., 2022).

## 4 Senescence: Triggers, pathways, markers, and its implications

Senescence is from the Latin root word “senex” meaning “slowing old” (Dodig et al., 2019). Cellular senescence is a dynamic process that leads to permanent cell cycle arrest and the loss of healthy cells’ physiological functions and gaining of new ones, which are mainly accrued through the release of many factors (Coppé et al., 2010). These new activities have been collectively indicated as Senescence-Associated Secretory Phenotype (SASP) (Coppé et al., 2010). Subsequently, the semantic extension of the term SASP refers to the secretome of senescent cells. SASP contains many bioactive molecules, such as growth factors, anti-apoptotic factors, pro-inflammatory cytokines, chemokines, and modulators of extracellular environment. Collectively, these factors can promote senescence of normal cells via paracrine signaling and reinforce the senescence process through autocrine mechanism (Coppé et al., 2010).

### 4.1 Triggers of senescence and executive programs

**FIGURE 3:**
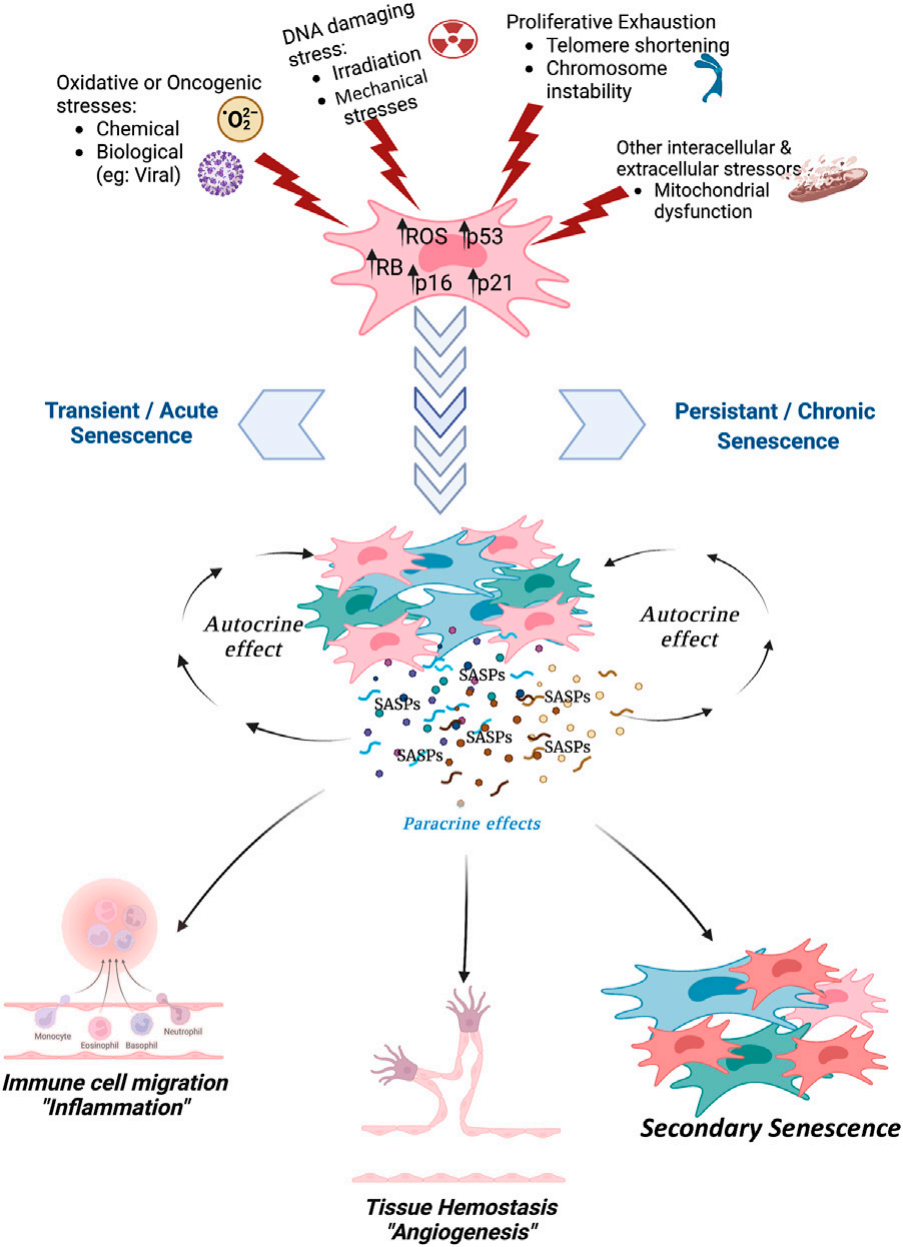
The figure depicts the several classes of stressors that can induce senescence: oxidative and oncogenic stresses; DNA damage; proliferative exhaustion; impairment of cellular function, such as mitochondrial and lysosomal activities. The activation of P53, RB, P21, and P16 pathways promote the onset of senescence. The senescent cells secrete SASP that can act in autocrine way to reinforce senescence and in paracrine way to induce secondary senescence in cells that were not directly hit by stressors. Figure was created with Biorender tools (https://www.biorender.com/) whose licence belongs to UG.

A number of nuclear stressors, as indicated in Figure 3, can promote the initiation and progression of cellular senescence and SASP *in vitro* as well as *in vivo* experimental models (Parikh et al., 2019; Zlotorynski, 2020; Martini and Passos, 2022; Takasugi et al., 2022).

The occurrence of cellular senescence is primarily, as far as we know, dependent on the activation of major tumor-suppression pathways controlled by P53, RB1, and P16-INK4A proteins (Coppé et al., 2010), which have been recently considered to be biomarkers to detect and monitor cellular senescence in culture and *ex-vivo* specimens (Figure 3). As the cellular senescence is triggered by various DNA stressors, the SASP begins to accumulate in the microenvironment of senescent cells or tissues, where these secreted biomolecules can exert either beneficial effects (i.e., tumor suppression) (Alessio et al., 2023) or deleterious effects such as pro-carcinogenic for secondary tumors, cancer relapse, and cancer chemotherapy-induced side effects (Demaria et al., 2017). SASP is also involved in the aging phenomena by promoting secondary senescence in healthy cells surrounding those directly hit by DNA stressors (paracrine effect of SASP) (Roger et al., 2021).

Given the paramount role of SASP in senescence and senescence-related phenomena, currently there are many studies on SASP obtained from various kinds of cells, including MSCs, fibroblasts, endothelial cells, liver stellate cells and epithelial cells of several human organs and murine MSCs, fibroblasts, and other cells of mouse origin as well as some cancer cells (Fausti et al., 2013; Loaiza and Demaria, 2016; Nishizawa et al., 2016; Abbadie et al., 2017; Caroti et al., 2017; Ferreira et al., 2018; Alessio et al., 2019b; Kotla et al., 2019; Dorronsoro et al., 2021; Kerschbaum et al., 2021; Schwartz et al., 2021; Zhang et al., 2021; Gerasymchuk et al., 2022; Yamada et al., 2022).

### 4.2 Markers to identify a senescent phenotype

The intrinsic dynamic nature of senescence and its variability, depending on cell type, cell status, and animal species, makes it difficult to establish a universal indicator to demonstrate the occurrence and progression of the senescence status. Currently, optimized and combined algorithms of different senescence-related biomarkers are the better option for the identification and isolation of senescent cells within a cell population. The main parameters used to identify senescent cells are: i) cell morphology; ii) evaluation of the expression of cell cycle regulators, such as P53, P21-CIP1, P16-INK4A, P38-MAPK, P14-ARF, at their transcriptional and translational levels; iii) detection of the expression of retinoblastoma gene family (RB1, RB2/P130, P107); iv) determination of senescence associated lysosomal β-galactosidase activity (SA-β-gal); v) identification of critical shorting of telomere DNA sequence length; and vi) lipofuscin detection using GL13 staining (Dimri et al., 1995; Debacq-Chainiaux et al., 2009; Georgakopoulou et al., 2013; Alessio et al., 2017; Evangelou et al., 2017; Salmonowicz and Passos, 2017; Son et al., 2019; Jannone et al., 2020; Chen et al., 2021; Ogrodnik, 2021; Guan et al., 2022; Wagner and Wagner, 2022; Weng et al., 2022). There are also website tools that may help in the identification of senescent cells. For instance, the Tumor Cellular Senescence Estimation Resource (TCSER, http://tcser.bmicc.org) can produce a cellular senescence score for quantification of senescence level and senescence related genes (Wang X. et al., 2022).

The SASP content and senescence–associated extracellular vesicles can also be major components of combined biomarkers for identifying senescent cells (Jeon et al., 2019; Basisty et al., 2020).

## 5 Senescence, aging, and aging related disorders

In youth many physiological functions have a spare capacity to better cope changes in environmental cues. With the age this spare capacity is lost due to the progressive impairment of many molecular mechanisms and physiological functions. These events lead to progressive loss of muscle mass, accumulation of adipose tissue and can contribute to onset of aging-related diseases (Al-Azab et al., 2022; Lu et al., 2022). A wide range of aging-related disorders are now reported from different corners of the world including osteoarthritis or cartilage degenerative diseases (Liu et al., 2022), reduced muscle regenerative processes (Lu et al., 2022), neurodegenerative diseases, cardiovascular diseases, cancer, immune dysfunction (Li et al., 2021), dementia, cataract, chronic obstructive pulmonary diseases (COPD), diabetes mellitus (Lancaster et al., 2018), and depression (Verhoeven et al., 2014).

As previously described, during aging, several biological and physical changes take place in mammalian organisms: immune aging (changes in number of T cells, loss of ability to respond to antigen, low grade inflammation) and cellular senescence are among the most important ones, though the biological aging or cellular senescence may not necessarily correlate with chronological age and are not used as typical hallmarks to monitor aging. However, since cellular senescence is associated with many types of aging related disorders, eliminating senescent cells and attenuation of their SASP components are currently attractive therapeutic strategies (Childs et al., 2015; Dodig et al., 2019; Lee et al., 2022).

*In vitro* experiments of MSC aging, the self-renewal, differentiation capacity, and stemness properties are always compromised with age, while the expression of P53, P21-CIP1, and P16-INK4A genes and the levels of reactive oxygen species (ROS) increase with chronological age (Kapetanos et al., 2021). The increased expression of the aforementioned cell cycle regulator genes may be directly associated with different spot DNA damages or accumulated reactive oxygen species that promote the process of cellular senescence. Progressive erosion of telomere length of proliferating cells *in vitro* triggers the DNA damage response (DDR) where the damage sensor ATM (ataxia telangiectasia mutated) kinase stabilizes the tumor suppressor protein (P53) and upregulates a cyclin-dependent kinase inhibitor (P21-CIP1), which in turn prevents cyclin-dependent kinase 2 (CDK2) mediated inactivation of RB1 and the subsequent entry of cells into the S-phase of the cell cycle (Childs et al., 2015; Mijit et al., 2020; Kumari and Jat, 2021). The ATM-P53-P21-CIP1 axis has also been implicated in other DNA damaging stresses including UV or gamma irradiation, chemotherapeutics, and RAS activations (Childs et al., 2015).

Despite the roles of senescence in maintaining normal tissue development, angiogenesis, tissue hemostasis, wound healing, and tumor inhibition, it is also implicated as one of the major causes of aging-associated disorders (McHugh and Gil, 2018). Hence, senescent cells are currently considered an interesting target for synthetic, natural, and biological therapeutic agents (Childs et al., 2015), which can be either senolytic (selectively targets and eliminates senescent cells) or senomorphic (inhibits the physiologic activities of senescent cells) (Martel et al., 2020). On the other hand, restoring senescence through tumor suppressing signaling or oncogene activation may cause cancer cells to respond to various chemotherapies (Childs et al., 2015), while persistent senescence induction may cause unexpected damage of the surrounding tissues through their secreted proteins, SASP (Childs et al., 2015; Watanabe et al., 2017).

## 6 Senescence profoundly affects the secretome composition of MSCs

During senescence, the MSC secretome, which contains factors that promote tissue repair and homeostasis, undergoes striking modifications in its composition and becomes SASP. When MSCs or their derivatives have to be used for therapeutic purposes, great attention must be paid to these changes.

We performed a concise literature review and compared the composition of SASP released by senescent MSCs (Table 1; Supplementary Files S1, S3) and secretome of healthy MSC populations (Table 2; Supplementary Files S2, S3).

**TABLE 1:** List of SASP factors identified in senescent mesenchymal stromal cells.

Immunomodulators (pro- and anti- inflammatory)	Cell cycle regulators	Metabolism (Catabolism/Anabolism)	ECM modulators	Growth and survival factors	Cell signaling	Others
CXCL10 , CXCL12 , MCP-1 (CCL2) , MCP-3, IL-6 , IL-8 (CXCL8) , IL-23, IL-1β , IL-1A, IL-7, IL-4, IL-15, CCL4, CCL20, CCL26, TLR2, TLR5, CXCR2, EGFR, GROβ(CXCL2), GRO(CXCL1), MIF, CD40ligand, CDKN2D, PSME1, PF4, GMCSF, RANTES(CCL5), IFNA2, Calreticulin	P53 , P21 , P16 , P27KIP1, pP38, P65, pP53, P107, P230, RB1, RB2/P130, TUBA1C, TUBB, Septin-2, Septin-9, RALA, ANG1, MDC	IDO-1, PTGS-2, PSMA1, PSMA3, PSMA5, PSMA6, PSMD2, NME1, UBE2V1, AK1, AK3	TIMP1, TIMP2, TIMP3, MMP1 , MMP2, MMP3 , MMP8, MMP10, MMP13, MMP14, SERPINEB2, FN1, SERPINE1(PAI-1) , CTSB, ICAM1, PLAT, ICAM3, A2M, ACTN1, ACTN4, ACTR2, VCL, EZR, MSN, MYH9, TLN1, COL1A1, COL1A2, COL3A1, COL4A2, COL8A1, COL6A1, COL6A2, COL6A3, COL12A1, CTSD, FBLN1, uPA, DPPIV, ADAMTS1, ADAMTS13, THBS1, EL3 (SPTB1), LUM, COMP, ACAN, FNDC1, CST3, ITM2B, LOXL1, AHNAK, SRGN, ABI3BP, TNC, BGN, FBN1, POSTN, FLNA	TGFβ-1, TGFβ-3, IGFBP7 , IGFBP6, IGFBP5, IGFBP4, IGFBP3, IGFBP2, HGF , PGF, PFG, PDGFRB , VEGFA , VEGFB , VEGFC , VEGFD , FGF7 , FGF2 , IGF1, GDF15 , NGF, EGF, ANG, AREG, KITLG, NRG1, EREG	PIGF, EGFR, TNFRSF1A, TNFRSF11B, TNFRSF10C, HSP90AA1, HSP90AB1, HSP90B1, HSPA4, HSPA5, HSPA8, HSPB1, HSPD1, HSPE1, PGE2, IRAK4, TAK1, IKKβ, GNAI2, GNB1, Activin A/INHBA	STC1 , PLAU, PLAUR, ILGST, PFN1, PFN2, IQGAP1, CFL1, RDX, ARPC1B, ARPC3, FASCIN, RPL5, RPL12, RPLP0, RPS12, RPS21, RPS3, RPS15A, FAS, BAX, ARHGDIA, GRP94, PPIB, PPIA, VIM

The list of secreted SASP factors listed in Table 1 is according to the following literatures (Severino et al., 2013; Özcan et al., 2015; O'Hagan-Wong et al., 2016; Özcan et al., 2016;Alessio et al., 2019a; Gnani et al., 2019; Alessio et al., 2020; Ratushnyy et al., 2020; Vassilieva et al., 2020; Kizilay Mancini et al., 2021; Kwon et al., 2021; Liao et al., 2021; Lehmann et al., 2022).

Note: In general, secreted factors from senescent MSCs were more abundant in number and various in type compared to its corresponding secretome from young/healthy MSCs. There are some common secreted factors secreted: those depicted in red bold font are either more expressed or secreted in senescent cultures compared with controls, while those in green bold font are either less expressed or secreted by senescent MSCs compared to the young/healthy MSC population.

**TABLE 2:** List of factors identified in healthy mesenchymal stromal cells (Secretome).

Immunomodulators (pro- and anti- inflammatory)	Cell cycle regulators	Metabolism (Catabolism/Anabolism)	ECM modulators	Growth and survival factors	Cell signaling	Others
IL-6, CXCL8 (IL-8), IL-1β, CCL2 (MCP-1), CXCL10, CXCL12	CCNA2, CDC20, CCNB2, CDCA5, KIAA0101, TOP2A, TYMS, AURKA, AURKB, NUSAP1, P^16INK4a^, P^21waf1^/CiP1, P^53^	HMOX1, PLCD1	THBS2, FN1, SERPINE1(PAI-1), NRCAM, NDNF, TMSB4X, SPON2, MMP1, MMP2, MMP3, MMP19	VEGF, HGF, TGFβ-1, IGFBP7, AAMP, MTDH, PDGFD, PDGFRL, LIF, ABI1, ANGPT1, CCBE1, ESM1, FGF2, FGF7, NAA15, PTN, VEGFA, TGF-β2, PDGF-BB,	TNFRSF12A, SFRP4, RIC8A, SFRP1, NOTCH3, RASA1, CRIM1, ENG, GDF15, JAG1, EFNB2, NRxN3, YWHAG, YWHAH, SFN, HSPH1, HSPD1, HSPA8, NEDD4, TSG101	PLAU, PLAUR, OG1, ELMO2, ADD1, STC1, KAT6A, AKT1, TMED2,

According to the literatures: (Infante and Rodríguez, 2018; Kehl et al., 2019; Grigorieva et al., 2021; Voskamp et al., 2021; Wang et al., 2022a).

This comparison may be of interest for the reasons we previously discussed, but it has its own intrinsic limits.

1) MSCs from different sources and animal species may differ in their healthy secretome and SASP composition;
2) Senescence induced by different stressors may produce distinct SASPs;
3) Senescence is a dynamic process, SASP composition of early senescent cells differs from that of late senescent cells;
4) Cited studies did not utilize the same methods to identify the factors present in secretome. Some studies performed the western blot analysis, others used dot blots for cytokine detection, and others performed liquid chromatography and mass spectrometry (LC-MS/MS). In some cases, researchers also detected the transcripts that are associated with factors present in SASP.

The most common sources of senescent MSCs were human and mouse bone marrow and adipose tissue, human umbilical cord, and human desquamated endometrium tissue, in which cellular senescence was induced using various genotoxic agents. Whereas healthy or non-senescent MSCs were originated from human bone marrow, adipose tissue, and umbilical cords (Wharton’s Jelly).

The literature included in the review employed several senescence-inducing agents such as H_2_O_2_, x-ray, and gamma-ray irradiations, extensive cultivation, exogenous factors: IGF-I, IGF-II, and IGFBP-4 (insulin-like growth factor I, insulin-like growth factor II, insulin-like growth factor binding protein 4), advanced glycation end products (AGE), WNT3A, MSC-derived exosomes, and Doxorubicin treatment (Supplementary File S1). In the literature we reviewed, the secretory phenotypes (which will be discussed in detail below) of senescent and healthy MSCs were detected using qRT-PCR, Western Blot, high resolution MS, LC-MS/MS, proteome profiler assay, immunoassay, inflammatory cytokine array, ELISA, and whole transcriptomic analysis (Supplementary Files S1, S2).

Large fractions of secreted proteins were observed in various functional groups of proteins of senescent MSCs (Table 1). In particular, secreted proteins in the panels of extracellular matrix (ECM) modulators, immunomodulators (pro- and anti-inflammatory), and cytoskeleton groups of SASPs from the senescent MSCs were more abundant relative to the corresponding proteins in secretome of healthy MSCs (Table 2). On the other hand, growth factors and cell cycle, and signaling proteins were elevated in the secretome of healthy MSCs compared to other groups of proteins of the same cell population (Table 2) while these secreted proteins remained unchanged in the counterparts of senescent MSCs. For the sake of clarity, the components identified in healthy MSC secretomes and in the SASP of senescent MSCs were grouped into 7 classes. This classification, even if it is arbitrary, may help in identifying factors that are exclusively present either in healthy or in aged cells.

In detail, healthy MSCs release factors involved in many biological processes, which have roles in preserving the tissue’s healthiness. Some released proteins are regulators of chondrogenesis, osteogenesis, adipogenesis, and angiogenesis (Özcan et al., 2016; Ayaz-Guner et al., 2020; Acar et al., 2021). Of note, bone marrow derived MSC secretome contains factors promoting the growth and the differentiation of glia and neurons. Other factors have immunomodulation activities and are also implicated in chemotaxis and migration of immune cells. Other secretome components play a key role in neutralizing toxic substances and drugs (detoxification activity) as well as counteracting reactive oxygen species (ROS) through many factors involved in oxidation-reduction reactions. Many factors released by MSCs play roles in the remodeling of ECM through the reshaping of glycosaminoglycans and other ECM components. The MSCs also release regulators of key cellular activities, such as synthesis and degradation of proteins and nucleic acids, regulators of metabolism, and modulators of endoplasmic reticulum stress (Özcan et al., 2016; Ayaz-Guner et al., 2020; Acar et al., 2021).

Senescence almost completely abolishes the release of these “protective factors” involved in tissue repair and homeostasis. The SASP of senescent MSCs still contains factors involved in ECM remodeling, regulation of metabolic processes, ox-redox- and immuno-modulators, regulators of synthesis and degradation of proteins and nucleic acids. Nevertheless, these factors are committed to the acquisition of the typical features of senescent cells and the loss of MSC original functions (Özcan et al., 2016; Ayaz-Guner et al., 2020; Acar et al., 2021).

Gene families of P53, RB, CDK inhibitors, growth factors, IGFBPs (insulin-like growth binding factors), prostaglandins, pro-inflammatory cytokines, chemokines, proteases, receptor proteins, cytoskeletal proteins, heat shock proteins, ribosomal proteins, proteasome activators, proangiogenic and angiogenesis related proteins, ECM proteins, fibronectin, collagens, actin binding proteins, SERPIN, and trans-Golgi network (TGN) proteins were identified from SASPs of senescence induced MSC cells (Table 1). The P53, RB, and CDK inhibitors, such as P16-INK4A, P27, and P21-CIP1, are primary genes that are activated under the cellular pathways responding to several DNA damaging stressors. Hence, promotors of these genes endorse their transcription and translation of effector proteins that can change the molecular functions of the cells themselves (autocrine effect) and overall tissue microenvironment (paracrine effect), propagating the stress responses from the senescent cells to the neighboring cells (Figure 3).

Upregulated expressions of these genes and detection of their downstream products in conditioned media of senescent cell populations usually correlated with the alteration of cell cycles, growth arrest, and occurrence of senescent cells in the heterogeneous population of MSC cells (Mijit et al., 2020; Kumari and Jat, 2021). The presence of increased levels of CDK inhibitors (P16-INK4A and P21-CIP1) in response to DNA damaging stresses maintain the de-phosphorylation of RB family proteins. This arrests cell cycle (Ohtani, 2022; Takasugi et al., 2022) and consequently leads to cellular senescence.

The senescence-associated secretory phenotype (SASP) is comprised of pro-inflammatory cytokines, growth factors, and their binding proteins; ECM components and enzymes may establish inflammatory, immunosuppressive, and catabolic microenvironment of the extracellular matrix of the tissues that lead to deleterious effects such as pro-carcinogenesis, tumorigenesis, and metastasis of the already established tumors (Basisty et al., 2020; Mavrogonatou et al., 2020) or to beneficial effects, such as alarming signals and activation of the tissue repairing machineries (Coppé et al., 2010).

Among the pro-inflammatory cytokines, interleukin-6 (IL-6) has been shown to be correlated with the senescence of different cell types including MSCs, keratinocytes, melanocytes, monocytes, fibroblasts, and epithelial cells, which appears to be secreted by persistent DNA-damaging signaling pathways. It directly affects the neighboring cells in the given tissues where it can adhere to GP80 and GP130 IL-6 receptors of epithelial and endothelial cells of experimental models (Coppé et al., 2010). As a consequence, JAK (Janus tyrosin kinase)/STAT (signal transducers and activators of transcription) and other signaling pathways can be activated inducing a self- and cross-reinforced senescence and strengthening tumorigenic capabilities of some cancer cell lines (Kojima et al., 2013; Ortiz-Montero et al., 2017; Taher et al., 2018; Li et al., 2020).

The growth factors and their inhibiting binding proteins are also components of senescence-associated secretory phenotypes (SASP) in the reviewed literature, where their altered representations were correlated to the occurrence of replicative or acute cellular senescence (Table 1).

For instance, VEGFB (vascular endothelial growth factor B) is among the growth factors that was upregulated in WNT3A induced MSC senescence. This factor may facilitate cancer development through promoting vascular angiogenesis of growing tumors (Coppé et al., 2006). The secretory profile of other growth factors like EGF (epidermal growth factor), HGF (hepatic growth factor), FGF (fibroblast growth factor), NGF (neural growth factors), and growth factor inhibitors: IGFBP-2, 3, 4, 5, 6, and 7 (Box 1), also contributed to the autocrine/paracrine effects of senescent cells exerted on their microenvironment of different senescent endothelial, epithelial, and fibroblast cells (Coppé et al., 2010; Zhao et al., 2020).

The extracellular roles of IGFBP-3, IGFBP-4, IGFBP-5, and IGFBP-7 have previously shown autocrine/paracrine senescence effects on i) human endometrial MSCs, ii) human umbilical endothelial cells, iii) BRAF-positive human primary fibroblasts, and iv) melanocytes, respectively. Increased levels of IGFBPs have been detected in the bloodstream of both irradiated mice and humans, and this may correlate with the release of these factors from senescent MSCs and other cell types. Unlike the other IGF binding protein species, IGFBP-6 overexpression leads to increased cellular life span and proved to be a negative regulator of cellular senescence in human fibroblasts (Kim et al., 2007; Wajapeyee et al., 2008; Micutkova et al., 2011; Severino et al., 2013; Alessio et al., 2020; Vassilieva et al., 2020).

The matrix metalloproteinase (MMP) enzymes are among the SASP components of senescence-induced MSCs (Table 1). These are the most important families of Zinc-dependent proteases involved in the functions and control of ECM, whose alterations can contribute to cellular senescence, the aging process, and aging-related disorders (Freitas-Rodríguez et al., 2017). Aging, replicative, or stress induced cellular senescence of MSCs or other stem cells alters the expressions and functions of MMPs that may affect the self-renewal capacity of stem cells, i. e, stemness characteristics, irreversibly degrade the ECM components, and shed cell surface receptors (Freitas-Rodríguez et al., 2017; Levi et al., 2020).

In addition to the degradation of ECM components, collagens in particular, MMPs can signal via cleavage and activation of different proteinase activated receptors (PARs), which triggers the establishment of pro-inflammatory conditions in Lithium-induced senescent endothelial cells (Struewing et al., 2009). In combination with TGF-β1, the MMP family can be sufficient to induce fibroblast senescence and consequent cancer promotion (Gabasa et al., 2021). Consistent upregulation of MMPs secreted by senescent fibroblast cells regulates soluble factors in the SASP, including CXCL/CCL family members of cytokines originating from surrounding neighboring cells, such as leukocytes, tumor cells, endothelial cells, and other cells (Coppé et al., 2010), that make it more complicated to understand the molecular signatures and functions of SASP in the extracellular microenvironment.

## 7 Conclusion

The analysis of MSC secretome is important either for the MSC transplants and for the therapeutic use of secretome. Indeed, the secretome of MSCs, which is the main mechanism of their therapeutic activity, loses its beneficial functions and acquire negative pro-inflammatory and pro-aging activities when MSCs become senescent.

Secreted molecules of senescence-induced MSCs including pro-inflammatory cytokines, chemokines, growth factors, insulin-like growth factor binding proteins, serine proteinase inhibitors, matrix metalloproteinases, proteasomes, and extracellular matrix proteins were the most frequently reported secretome components. These factors could be used as biomarkers of senescence-induced conditions in aging-related or chronic diseases and they may be also useful for preliminary evaluation of MSC samples and their derivatives (extracellular vesicles, secretomes) that have to be used in cell therapy. Indeed, inter-donor variability, *ex vivo* proliferation, *in vitro* cryopreservation (Galipeau, 2013), stem cell quantity and quality such as the percentage of stem/progenitor cells, surface marker expression, clonal expansion, multi-differentiating capacity, and the presence of senescent cells may also contribute to the unsuccessful MSC clinical trials.
