# Supplementary material for: Senescence induces fundamental changes in the secretome of mesenchymal stromal cells (MSCs): implications for the therapeutic use of MSCs and their derivates
Source: Front Bioeng Biotechnol. 2023 May 9;11:1148761. doi: 10.3389/fbioe.2023.1148761 (PMC10203235; doi:10.3389/fbioe.2023.1148761)
Supplement: Supplementary file 2 [file Table1.DOCX]

Supplementary Materials

*The senescence induces fundamental changes in the secretome of mesenchymal stromal cells: implications for their therapeutic use*

**Yesuf Siraj^1,2^, Umberto Galderisi^1,3,4*^, Nicola Alessio^1^**

^1^Department of Experimental Medicine, Luigi Vanvitelli Campania University, Naples, Italy

^2^Department of Medical Laboratory Sciences, School of Health Sciences, College of Medicine and Health Sciences, Bahir Dar University, Bahir Dar, Ethiopia

^3^Department of Biology, Faculty of Science, Erciyes University, Kayseri, Turkey

^4^Center for Biotechnology, Sbarro Institute for Cancer Research and Molecular Medicine, Temple University, Philadelphia, PA, United States

# Supplementary file 1:

**Supplementary file 1: SASP secreted from senescent MSCs**

| **Article No.** | **Author, YYYY**  **(Ref.)** | **Source of MSCs** | **Type of Senescence** | **Stressor** | **Experimental Cells/Model** | **Detected SASP genes/mRNAs/proteins** | **Class of proteins** | **SASP status** | **Method used** |
| --- | --- | --- | --- | --- | --- | --- | --- | --- | --- |
| 1. | ([Alessio et al., 2019a](#_ENREF_3)) | Human Bone Marrow | Acute and Replicative | H_2_O_2_ & X-ray  and Repeated Passaging | PNT2 | P^53^ | P53 family | Increased | Western Blot |
|  |  |  |  |  |  | RB2/P^130^ | RB family | Increased |  |
|  |  |  |  |  |  | P^107^ | RB family | Decreased |  |
|  |  |  |  |  |  | P^27KIP1^ | CDK Inhibitors | Increased |  |
|  |  |  |  |  | PC3 | RB1 | RB family | Decreased |  |
|  |  |  |  |  |  | RB2/^P130^ | RB family | Increased |  |
|  |  |  |  |  |  | P^107^ | RB family | Increased |  |
| 2. | ([Alessio et al., 2020](#_ENREF_6)) | Human Bone Marrow | Acute and Replicative | H_2_O_2_ & X-ray  and Extensive Cultivation | - | IGFBP-4 | IGFBP family | Increased | Western Blot |
|  |  |  |  |  |  | PGE-2 | Prostaglandin |  |  |
|  |  |  | Acute | Exogenous IGF-I/IGF-II/IGFBP-4 | - | RB, RB2, P^107^ | RB family | Activated |  |
|  |  |  |  |  |  | P^27^, P^21^, P^16^ | CDK Inhibitors |  |  |
|  |  |  |  |  |  | P^53^ | P53 family |  |  |
|  |  | Mice Bone Marrow (Irradiated) | Early | X-ray irradiation | - | P^53^ | P53 family | Highly Increased |  |
|  |  |  |  |  |  | P^27^ | CDK Inhibitor |  |  |
|  |  |  |  |  |  | RB | RB family | Moderately Increased |  |
|  |  |  |  |  |  | P^16^, P^21^ | CDK Inhibitors |  |  |
|  |  | Mice Bone Marrow (IGFBP-4 treated) | Early | Exogenous  IGFBP-4 | - | RB | RB family | Highly Increased |  |
|  |  |  |  |  |  | P^53^ | P53 family |  |  |
|  |  |  |  |  |  | P^27^, P^21^, P^16^ | CDK Inhibitors | Moderately Increased |  |
| 3. | ([Gnani et al., 2019](#_ENREF_41)) | Human Bone Marrow | Replicative | Repeated Passaging | HSPC | CXCL10 | Chemokine | Decreased | qRT-PCR |
|  |  |  |  |  |  | IDO-1, PTGS-2, TGFβ-1 |  | No change |  |
|  |  |  |  |  |  | MCP1, IL-6, IL-8, Groβ, CCL4 | Pro-inflammatory cytokine | Robustly Increased |  |
| 4. | ([Kizilay Mancini et al., 2021](#_ENREF_57)) | Adipose Tissues (T2DM + Atherosclerosis) | AGE induced Acute senescence | Advanced Glycolytic End (AGE) | MSC | IL-6, IL-8(CXCL8), MCP-1 | Cytokine | Increased | ELISA |
|  |  |  |  |  |  | IRAK4, TAK1, IKKβ, P^65^ | Protein Kinase |  |  |
|  |  |  |  |  |  | IL-6, IL-8(CXCL8), MCP-1 (CCL2) | Cytokine | Increased | Western Blot |
| 5. | ([Kwon et al., 2021](#_ENREF_62)) | Human Umbilical Cord Blood | Replicative | Repetitive Sub-culturing | Hetrogenous MSC (>10µm) | P^16^, P^21^ | CDK Inhibitors | Increased | Western Blot |
|  |  |  |  |  |  | p-P^38^ | MAPKs |  |  |
|  |  |  |  |  |  | p-P^53^ | P53 family |  |  |
|  |  |  |  |  | Small MSC (<10µm) | GRO, IL-8, MCP-1, IL-6, IL-23 | Cytokine | Lower Secretion | Inflammatory Cytokine Array with 40 spots |
|  |  |  |  |  |  | MIF | MIF family |  |  |
|  |  |  |  |  |  | CD40ligand | TNF superfamily |  |  |
|  |  |  |  |  |  | TLR2, TLR5 | TLR family | Lower Expression | qPCR |
|  |  |  |  |  |  | CXCR2 | GPCR family |  |  |
| 6. | ([Lehmann et al., 2022](#_ENREF_65)) | Human Bone Marrow | Acute | WNT3A | MSC | IL-6, IL-1B, IL-1A, IL-7 | Cytokine | Down regulated | Whole Transcriptomic Analysis |
|  |  |  |  |  |  | IL-15 | Cytokine | Upregulated |  |
|  |  |  |  |  |  | CCL2, CCL26, CCL20 | Chemokine | Down regulated |  |
|  |  |  |  |  |  | MIF | Chemokine | Upregulated |  |
|  |  |  |  |  |  | IGFBP7, HGF, VEGFA, VEGFC, FGF7, ANG, CXCL12, STC1, IGFBP2, GDF15, FGF2, AREG, PIGF, VEGFD, PGF, IGFBP3, IGFBP4, KITLG | Growth Factors and IGFBPs | Down regulated |  |
|  |  |  |  |  |  | NGF, NRG1, VEGFB, IGFBP6, EREG, EGF | Growth Factors and IGFBPs | Upregulated |  |
|  |  |  |  |  |  | TIMP1, MMP14, MMP13, MMP1, CTSB, MMP3 | MMPs and Protease | Down regulated |  |
|  |  |  |  |  |  | TIMP2 | TIMP family | Upregulated |  |
|  |  |  |  |  |  | MMP10 | MMPs |  |  |
|  |  |  |  |  |  | PLAU, PLAUR, TNFRSF1A, SERPINEB2, TNFRSF10C, ICAM1, PLAT, ILGST, ICAM3 | Shed Receptor | Dow regulated |  |
|  |  |  |  |  |  | SERPINE1, TNFRSF11B, EGFR, FAS | Shed Receptor | Upregulated |  |
| 7. | ([Liao et al., 2021](#_ENREF_69)) | Human Umbilical Cord (UC-MSC) | Replicative | MSC-derived Exosome | UC-MSC | CDKN2D, P^16INK4a^ | CDK Inhibitor | Reduced expression | RT-qPCR |
|  |  |  |  |  |  | IL-6, IL-8 | Cytokine |  |  |
| 8. | ([O'Hagan-Wong et al., 2016](#_ENREF_88)) | Human Adipose Tissue | Acute | Gamma Irradiation | A-MSC | IL-6, IL-8 | Cytokine | Increased | ELISA |
| 9. | ([Özcan et al., 2016](#_ENREF_92)) | Human Adipose Tissue and Bone Marrow | Acute and Replicative | Oxidative Stress, Doxorubicin, X-ray and Replicative exhaustion | A-MSC | A2M | A2M family | Over represented | LC-MS/MS |
|  |  |  |  |  |  | MMP2 | MMP family |  |  |
|  |  |  |  |  |  | TIMP1, TIMP2 | TIMP family |  |  |
|  |  |  |  |  |  | ACTN1, ACTN4, ACTR2 | Actin family |  |  |
|  |  |  |  |  |  | TUBA1C, TUBB | Tubulin superfamily |  |  |
|  |  |  |  |  |  | VCL | Vinculin |  |  |
|  |  |  |  |  |  | EZR, MSN, MYH9 | ERM (ezrin-radixin- moesin) protein |  |  |
|  |  |  |  |  |  | PFN1 | Profilin family |  |  |
|  |  |  |  |  |  | TLN1 | Tallin |  |  |
|  |  |  |  |  |  | COL1A1, COL1A2, COL3A1, COL6A2 | Collagen |  |  |
|  |  |  |  |  |  | FN1 | Fibronectin |  |  |
|  |  |  |  |  |  | IGFBP3 | IGFBP |  |  |
|  |  |  |  |  |  | SERPINE1 | SERPINE |  |  |
|  |  |  |  |  |  | HSP90AA1, HSP90AB1, HSP90B1, HSPA5, HSPA8, HSPB1, HSPD1 | Heat shock protein family |  |  |
|  |  |  |  |  |  | PSMA1, PSMA3, PSMA5, PSMA6 | Proteasome alpha-type subunit |  |  |
|  |  |  |  |  |  | GNAI2 | G-protein alpha |  |  |
|  |  |  |  |  | BN-MSC | A2M | A2M family | Over represented |  |
|  |  |  |  |  |  | MMP2 | MMP family |  |  |
|  |  |  |  |  |  | TIMP2 | TIMP family |  |  |
|  |  |  |  |  |  | ACTN1, ACTN4, ACTR2, ARPC3 | Actin-related protein |  |  |
|  |  |  |  |  |  | IQGAP1 | IQGAP family |  |  |
|  |  |  |  |  |  | TUBB | Tubulin superfamily |  |  |
|  |  |  |  |  |  | VCL | Vinculin |  |  |
|  |  |  |  |  |  | NME1 | Nucleoside di-phosphate kinase |  |  |
|  |  |  |  |  |  | CFL1 | Cofilin |  |  |
|  |  |  |  |  |  | FN1 | Fibronectin |  |  |
|  |  |  |  |  |  | PFN1 | Profilin family |  |  |
|  |  |  |  |  |  | MSN, MYH9, RDX | ERM (ezrin-radixin- moesin) protein |  |  |
|  |  |  |  |  |  | COL1A1, COL1A2, COL3A1 | Collagen |  |  |
|  |  |  |  |  |  | IGFBP3 | IGFBP |  |  |
|  |  |  |  |  |  | SERPINE1 | SERPINE |  |  |
|  |  |  |  |  |  | HSP90AA1, HSP90AB1, HSP90B1, HSPA4, HSPA5, HSPA8, HSPB1, HSPD1 | Heat shock protein family |  |  |
|  |  |  |  |  |  | PSMA5 PSMB1 PSMB4 PSMB5 PSMB6 | Proteasome alpha-type subunit |  |  |
| 10. | ([Özcan et al., 2015](#_ENREF_93)) | Human Bone Marrow | Acute and Replicative | H_2_O_2_, Doxorubicin and Replicative | MSC  (Naïve secretome) | ARPC1B, ARPC3, ARPC5, ACTR2 | Actin-related protein | Over Represented | LC-MS/MS |
|  |  |  |  |  |  | CTSD | Cathepsin proteins |  |  |
|  |  |  |  |  |  | GNB1 | G Proteins |  |  |
|  |  |  |  |  |  | CSTB | Cystatin superfamily |  |  |
|  |  |  |  |  |  | PLIN3 | Perilipin family |  |  |
|  |  |  |  |  |  | BAX | Bcl-2 family |  |  |
|  |  |  |  |  |  | FN1 | Fibronectin |  |  |
|  |  |  |  |  |  | PFN2 | Profilin family |  |  |
|  |  |  |  |  |  | FBLN1 | Fibulin |  |  |
|  |  |  |  |  |  | RPS3 | S3P family of ribosomal proteins |  |  |
|  |  |  |  |  |  | UBE2V1 | Ubiquitin-conjugating enzyme |  |  |
|  |  |  |  |  |  | AK3, AK1 | adenylate kinase family |  |  |
|  |  |  |  |  |  | RPS15A | S8P family of ribosomal proteins |  |  |
|  |  |  |  |  |  | PSMD2 | 26S proteasome regulatory complex |  |  |
|  |  |  |  |  |  | RPL5, RPL12, RPLP0, RPS12, RPS21 | Ribosomal proteins |  |  |
|  |  |  |  |  |  | PSME1 | Proteasome activator superfamily |  |  |
|  |  |  |  |  |  | HSPE1 | Heat shock protein family |  |  |
|  |  |  |  |  |  | FASIN | Fascin family |  |  |
|  |  |  |  |  | Myeloma cells | SEPT2, SEPT9 | septin family | Over Represented |  |
|  |  |  |  |  |  | ARHGDIA | Rho protein GDP-dissociation inhibitor |  |  |
|  |  |  |  |  |  | RALA | Small GTPase |  |  |
|  |  |  |  |  |  | TLN1 | Tallin |  |  |
| 11. | ([Ratushnyy et al., 2020](#_ENREF_96)) | Human Adipose Tissue | Replicative | Long term cultivation | MSC, HUVEC, OVO | MMP-8, IL-1B, Ang-1, PF4, uPA, DPPIV, Activin A, GM-CSF, IL-8, MCP-1 | Angiogenesis related proteins | Increased | Proteome Profiler Array |
|  |  |  |  |  |  | PAI-1 | SERPIN | Decreased |  |
|  |  |  |  |  |  | IL-6, IL-8 | Interleukin | Increased |  |
|  |  |  |  |  |  | MCP-1, GRO (CXCL1) | Chemokine |  |  |
|  |  |  |  |  |  | RANTES(CCL5), FGF2, IP10, MDC, IFNa2, IL-4, MCP-3 | Cytokine | Increased | Immunoassay |
|  |  |  |  |  |  | IGF1, MMP1, TGFB3, PFG, PDGFRB | Pro-angiogenic activities | Down regulated | RT-qPCR |
|  |  |  |  |  |  | MMP8, ADAMTS13, THBS1, TGFBI, IGFBP3, TIMP3, uPA, TIMP2, ADAMTS1 | Anti-angiogenic activities | Upregulated |  |
| 12. | ([Severino et al., 2013](#_ENREF_103)) | Human Bone Marrow | Replicative | Repeated Passaging to P10 | CM of Young MSC (P1) | Calreticulin | calcium-binding proteins | Over represented | LC-MS/MS |
|  |  |  |  |  |  | Collagen alpha-1(VIII) chain | short chain collagen family |  |  |
|  |  |  |  |  |  | EL3 | ECM protein |  |  |
|  |  |  |  |  |  | Endoplasmin | Reticuloplasmin |  |  |
|  |  |  |  |  |  | HSP90-beta | heat-related proteins |  |  |
|  |  |  |  |  |  | Lumican (LUM) | small leucine-rich proteoglycan (SLRP) |  |  |
|  |  |  |  |  |  | Peptidyl-prolyl cis-trans isomerase B | peptidyl-prolyl cis-trans isomerase |  |  |
|  |  |  |  |  | CM of Young MSC (P1) | Calcium-binding protein | Calcium Binding Protein family | Over represented |  |
|  |  |  |  |  |  | Cartilage oligomeric matrix protein | Thrombospondin family |  |  |
|  |  |  |  |  |  | Aggrecan core protein | Lectican family |  |  |
|  |  |  |  |  |  | Cystatin-C | Cystatin family |  |  |
|  |  |  |  |  |  | Fibronectin type III domain-containing protein 1 | Fibronectin type III domain |  |  |
|  |  |  |  |  |  | IGFBP-5 | IGFBP |  |  |
|  |  |  |  |  |  | Integral membrane protein 2B (ITM2B) | type 2 integral membrane protein (ITM2) |  |  |
|  |  |  |  |  |  | Lysyl oxidase homolog 1 (LOXL1) | Lysyl oxidase family |  |  |
|  |  |  |  |  |  | Neuroblast differentiation-associated protein (AHNAK) | Plasma membrane-associated proteins |  |  |
|  |  |  |  |  |  | Peptidyl-prolyl cis-trans isomerase A | Immunophilins |  |  |
|  |  |  |  |  |  | Serglycin | serglycin family |  |  |
|  |  |  |  |  |  | Target of Nesh-SH3 | ABI Family |  |  |
|  |  |  |  |  |  | Tenascin | Tenascin family |  |  |
|  |  |  |  |  | Ratio of CM-P10 to CM-P1 of MSC | Biglycan | small leucine-rich proteoglycan family | Over represented |  |
|  |  |  |  |  |  | Collagen alpha-1(VI) chain | Collagen VI family |  |  |
|  |  |  |  |  |  | Collagen alpha-2(VI) chain | Collagen VI family |  |  |
|  |  |  |  |  |  | Collagen alpha-3(VI) chain | Collagen VI family |  |  |
|  |  |  |  |  |  | Fibrillin-1 | Fibrillin family |  |  |
|  |  |  |  |  |  | Periostin | Fasciclin family |  |  |
|  |  |  |  |  |  | Collagen alpha-1(XII) chain | Collagen XII family | Over represented |  |
|  |  |  |  |  |  | Collagen alpha-2(I) chain | Collagen I family |  |  |
|  |  |  |  |  |  | Collagen alpha-2(IV) chain | Collagen IV family |  |  |
|  |  |  |  |  |  | Filamin-A | Actin-binding proteins |  |  |
|  |  |  |  |  |  | IGFBP-4 | IGFBP family |  |  |
|  |  |  |  |  |  | IGFBP-7 | IGFBP family |  |  |
|  |  |  |  |  |  | Vimentin | Intermediate filament family |  |  |
| 13. | ([Vassilieva et al., 2020](#_ENREF_114)) | Human Desquamated Endometrium | Acute Senescence | H_2_O_2_ | MSC | IGFBP-3 | IGFBP family | Increased | High resolution MS |
|  |  |  |  |  |  | PAI-1 | SERPIN |  |  |
|  |  |  |  |  |  | P^53^ | P^53^ family | Increased | Western Blot |
|  |  |  |  |  |  | PAI-1 | SERPIN |  |  |
|  |  |  |  |  |  | IGFBP-3 | IGFBP family | Up regulated | qRT-PCR |
|  |  |  |  |  |  | P^230^ | Trans-Golgi Network (TGN) protein family |  |  |
