# Supplementary material for: Senescence induces fundamental changes in the secretome of mesenchymal stromal cells (MSCs): implications for the therapeutic use of MSCs and their derivates
Source: Front Bioeng Biotechnol. 2023 May 9;11:1148761. doi: 10.3389/fbioe.2023.1148761 (PMC10203235; doi:10.3389/fbioe.2023.1148761)
Supplement: Supplementary file 3 [file Table2.DOCX]

***Supplementary Materials***

***The senescence induces fundamental changes in the secretome of mesenchymal stromal cells: implications for their therapeutic use***

**Yesuf Siraj^1,2^, Umberto Galderisi^1,3,4*^, Nicola Alessio^1^**

^1^Department of Experimental Medicine, Luigi Vanvitelli Campania University, Naples, Italy

^2^Department of Medical Laboratory Sciences, School of Health Sciences, College of Medicine and Health Sciences, Bahir Dar University, Bahir Dar, Ethiopia

^3^Department of Biology, Faculty of Science, Erciyes University, Kayseri, Turkey

^4^Center for Biotechnology, Sbarro Institute for Cancer Research and Molecular Medicine, Temple University, Philadelphia, PA, United States

**Supplementary file 2:**

**Supplementary file 2: *In vitro* secretome of healthy / non senescent MSCs**

| **Article No.** | **Author, YYYY**  **(Ref.)** | **Source of MSCs** | **Type of Senescence** | **Treatment** | **Experimental Cells/Model** | **Detected Secretome** | **Family** | **Status** | **Method used** |
| --- | --- | --- | --- | --- | --- | --- | --- | --- | --- |
| 1. | ([Grigorieva et al., 2021](#_ENREF_43)) | Human Subcutaneous Adipose Tissue | - | Platelet derived growth factor (PDGF) | MSC and HUVEC | CCNA2, CCNB2, CDC20, CDCA5, KIAA0101, TOP2A, TYMS, AURKA, AURKB, NUSAP1 | Proliferation and cell cycle | Upregulated expression | Transcriptomic analysis |
|  |  |  |  |  |  | P^16INK4a^, P^21waf1^/CiP1, P^53^ | CDK Inhibitors | Down regulated |  |
|  |  |  |  |  |  | PLAU, PLAUR, MMP-1, SERPINE1(PAI-1) | Cell Migration Genes | Down regulated |  |
|  |  |  |  |  |  | CCL8 (IL-8), CCL2 (MCP-1), CXCL10, CXCL12, IL-6, THBS2 | Chemokine | Down regulated |  |
|  |  |  |  |  |  | IL-6 | Chemokine | Decreased | ELISA |
|  |  |  |  |  |  | MCP-1 | Chemokine | No change |  |
|  |  |  |  |  |  | PAI-1 | SERPIN | No change |  |
|  |  |  |  |  |  | VEGF | VEGF family | Increased |  |
|  |  |  |  |  |  | HGF | Plasminogen subfamily |  |  |
| 2. | ([Infante and Rodríguez, 2018](#_ENREF_47)) | Human Bone marrow | - | Prelamin A | MSCs | FN-1 | Fibronectin | Upregulated | LC-MS/MS |
|  |  |  |  |  |  | TGFβ-1 | TGF family |  |  |
|  |  |  |  |  |  | IGFBP-7 | IGFB superfamily |  |  |
|  |  |  |  |  |  | PAI-1 | SERPIN |  |  |
| 3. | ([Kehl et al., 2019](#_ENREF_53)) | Human Adipose Tissue | - | - | hA-MSC | HIMOX1 | Heme oxygenase | Over represented | LC-MS.MS |
|  |  |  |  |  |  | OG1 | Osteocalcin |  |  |
|  |  |  |  |  |  | CXCL12 | Chemokine |  |  |
|  |  |  |  |  |  | ELMO2 | ELMO proteins |  |  |
|  |  |  |  |  |  | NDNF | Neurotrophic factor |  |  |
|  |  |  |  |  |  | TNFRSF12A | TNF receptor family |  |  |
|  |  |  |  |  |  | NRCAM | L1 family of immunoglobulin |  |  |
|  |  |  |  |  |  | SFRP4 | SFRP family |  |  |
|  |  |  |  |  |  | TMSB4X | beta-thymosin |  |  |
|  |  |  |  |  |  | AAMP | immunoglobulin superfamily |  |  |
|  |  |  |  |  |  | MTDH | Metadherin ? |  |  |
|  |  |  |  |  |  | PDGFD | platelet-derived growth factor family |  |  |
|  |  |  |  |  |  | PFGFRL | FGFR family |  |  |
|  |  |  |  |  |  | RIC8A | Synembryn family |  |  |
|  |  |  |  |  |  | SFRP1 | SFRP family |  |  |
|  |  |  |  |  |  | SPON2 | Mindin-F-Spondin |  |  |
|  |  | Human Bone Marrow | - | - | hB-MSC | LIF | interleukin 6 class cytokine | Over represented |  |
|  |  |  |  |  |  | NOTCH3 | Notch family |  |  |
|  |  |  |  |  |  | PLCD1 | Phospholipase C |  |  |
|  |  |  |  |  |  | RASA1 | GAP1 family |  |  |
|  |  |  |  |  |  | CXCL12 | Chemokine |  |  |
|  |  |  |  |  |  | TMSB4X | beta-thymosin |  |  |
|  |  |  |  |  |  | TNFRSF12A | TNF receptor family |  |  |
|  |  |  |  |  |  | ABI1 | Abelson-interactor |  |  |
|  |  |  |  |  |  | ADD1 | cytoskeletal proteins |  |  |
|  |  |  |  |  |  | ANGPT1 | angiopoietin family |  |  |
|  |  |  |  |  |  | CCBE1 | ECM protein |  |  |
|  |  |  |  |  |  | CRIM1 | Growth factor receptor |  |  |
|  |  |  |  |  |  | ENG | TGF-β |  |  |
|  |  |  |  |  |  | ESM1 | PGs family |  |  |
|  |  |  |  |  |  | FGF2 | Fibroblast growth factor family |  |  |
|  |  |  |  |  |  | FGF7 |  |  |  |
|  |  |  |  |  |  | GDF15 | TGF- β |  |  |
|  |  |  |  |  |  | JAG1 | EGF-like domain |  |  |
|  |  |  |  |  |  | KAT6A | histone acetyltransferases |  |  |
|  |  |  |  |  |  | STC1 | Stanniocalcin |  |  |
|  |  | Human Umbilical Cord (Wharton’s Jelly) | - | - | hWJSC | AKT1 | serine/threonine-protein kinases | Over represented |  |
|  |  |  |  |  |  | EFNB2 | Ephrin (EPH) family |  |  |
|  |  |  |  |  |  | MMP19 | MMP family |  |  |
|  |  |  |  |  |  | NAA15 | N-terminal acetyltransferase A |  |  |
|  |  |  |  |  |  | NRxN3 | presynaptic cell adhesion proteins |  |  |
|  |  |  |  |  |  | PTN | pleiotrophin (PTN)/MK family |  |  |
|  |  |  |  |  |  | TMED2 | Transmembrane emp24 domain-containing protein |  |  |
|  |  |  |  |  |  | ABI1 | Abelson-interactor |  |  |
|  |  |  |  |  |  | ADD1 | cytoskeletal proteins |  |  |
|  |  |  |  |  |  | ANGPT1 | angiopoietin family |  |  |
|  |  |  |  |  |  | CCBE1 | ECM protein |  |  |
|  |  |  |  |  |  | CRIM1 | Growth factor receptor cysteine-rich domain superfamily |  |  |
|  |  |  |  |  |  | ENG | TGF-β |  |  |
|  |  |  |  |  |  | ESM1 | PGs family |  |  |
|  |  |  |  |  |  | FGF2 | Fibroblast growth factor family |  |  |
|  |  |  |  |  |  | FGF7 |  |  |  |
|  |  |  |  |  |  | GDF15 | TGF- β |  |  |
|  |  |  |  |  |  | JAG1 | EGF-like domain |  |  |
|  |  |  |  |  |  | KAT6A | histone acetyltransferases |  |  |
|  |  |  |  |  |  | STC1 | Stanniocalcin |  |  |
|  |  |  |  |  |  | AAMP | immunoglobulin superfamily |  |  |
|  |  |  |  |  |  | MTDH | Metadherin ? |  |  |
|  |  |  |  |  |  | PDGFD | platelet-derived growth factor family |  |  |
|  |  |  |  |  |  | PFGRL | FGFR family ? |  |  |
|  |  |  |  |  |  | RIC8A | Synembryn family |  |  |
|  |  |  |  |  |  | SFRP1 | SFRP family |  |  |
|  |  |  |  |  |  | SPON2 | Mindin-F-Spondin |  |  |
| 4. | ([Voskamp et al., 2021](#_ENREF_115)) | Human Bone Marrow | - | TWIST1 Silencing | MSC | CCL2, IL-1β | Chemokine | High level expression | Transcriptomic analysis |
|  |  |  |  |  |  | IL-6, MMP-3, VEGFA | Interleukin | No change |  |
|  |  |  |  |  |  | IL-8 | Interleukin | Low level expression |  |
| 5. | ([Wang et al., 2022a](#_ENREF_119)) | Fetal Bone Marrow | - | - | Bioreactor based secretion (MSC) | TGF-β2 | TGF- β | Increased | UHPLC MS |
|  |  |  |  |  |  | PDGF-BB | platelet-derived growth factor family |  |  |
|  |  |  |  |  |  | MMP-1, MMP-2 | MMP family |  |  |
|  |  |  |  |  |  | YWHAG, SFN, YWHAH | 14-3-3 protein family | Highly expressed | qPCR |
|  |  |  |  |  |  | HSPH1, HSPD1, HSPA8 | Heat Shock Protein |  |  |
|  |  |  |  |  |  | NEDD4 | Ubiquitin ligases |  |  |
|  |  |  |  |  |  | TSG-101 | ubiquitin-conjugating enzyme | High protein expression | Western Blot |
